# Magnetic compass of garden warblers is not affected by oscillating magnetic fields applied to their eyes

**DOI:** 10.1038/s41598-020-60383-x

**Published:** 2020-02-26

**Authors:** Julia Bojarinova, Kirill Kavokin, Alexander Pakhomov, Roman Cherbunin, Anna Anashina, Maria Erokhina, Maria Ershova, Nikita Chernetsov

**Affiliations:** 1 Department Vertebrate Zoology, St. Petersburg State University, 199034 St. Petersburg, Russia; 2 Sechenov Institute of Evolutionary Physiology and Biochemistry, Russian Academy of Sciences, 194223 St. Petersburg, Russia; 3 Spin Optics Lab., St. Petersburg State University, 198504 St. Petersburg, Russia; 4 Biological Station Rybachy, Zoological Institute of the Russian Academy of Sciences, 238535 Rybachy, Kaliningrad Region Russia; 5 Department Natural Science and Geography, Ilya Ulyanov State Pedagogical University, 432700 Ulyanovsk, Russia; 6 Department Vertebrate Zoology, Lomonosov Moscow State University, 119234 Moscow, Russia

**Keywords:** Chemical biology, Zoology

## Abstract

The magnetic compass is an important element of the avian navigation system, which allows migratory birds to solve complex tasks of moving between distant breeding and wintering locations. The photochemical magnetoreception in the eye is believed to be the primary biophysical mechanism behind the magnetic sense of birds. It was shown previously that birds were disoriented in presence of weak oscillating magnetic fields (OMF) with frequencies in the megahertz range. The OMF effect was considered to be a fingerprint of the photochemical magnetoreception in the eye. In this work, we used miniaturized portable magnetic coils attached to the bird’s head to specifically target the compass receptor. We performed behavioural experiments on orientation of long-distance migrants, garden warblers (*Sylvia borin*), in round arenas. The OMF with the amplitude of about 5 nT was applied locally to the birds’ eyes. Surprisingly, the birds were not disoriented and showed the seasonally appropriate migratory direction. On the contrary, the same birds placed in a homogeneous 5 nT OMF generated by large stationary coils showed clear disorientation. On the basis of these findings, we suggest that the disruption of magnetic orientation of birds by oscillating magnetic fields is not related to photochemical magnetoreceptors in their eyes.

## Introduction

The magnetic compass of migratory birds is based on a sensory modality which is lacking in humans (but see^1^). This is the reason why the use of a magnetic compass baffles the researchers. However, the existence of a magnetic compass system in birds has been well documented and is no longer challenged^2^. Apart from avian magnetic sense, there are reports on the use of a magnetic compass by mammals^3–5^, sea turtles^6,7^, amphibians^8–11^ and bony fishes^12^. Nevertheless, the data available by now do not reliably elucidate the basic mechanisms of compass magnetoreception: receptor cells have not been identified, mechanisms of signal transduction remain unknown and even the location of such receptors is questioned. In spite of a significant period of time which elapsed since the discovery of the magnetic compass in animals, its sensory mechanism remains obscure, even though a significant progress has been achieved in this field in the recent years^13^. From the basic biological perspective, the perception of the magnetic field remains the only sense for which the sensory mechanism and its location still remain unknown.

Currently, as applied to the magnetic compass of birds, the model of spin-dependent chemical reactions, also known as the radical pair model (RPM), is the most popular one^14–18^. According to this model, the primary biophysical detection of the magnetic field is provided by molecules of a photosensitive protein, cryptochrome, located in the retina of the bird’s eye. The yield of photochemical reactions in cryptochrome is sensitive to magnetic field^17^; furthermore, it depends on orientation of the molecule with respect to the field^18^. It is supposed that molecular axes of cryptochromes are aligned in accord with local orientation of the retina surface. Since the latter is curved, force lines of the geomagnetic field form different angles with cryptochrome molecules at different points of the retina. According to the model proposed in ref. ^15^,this would allow birds to perceive the geomagnetic field as a pattern superimposed on the visual image. In a sense, they could literally ‘see’ the magnetic field^15^. The radical pair model predicts that oscillating electromagnetic field in the lower megahertz range (1–100 MHz) can disrupt the magnetic compass due to the electron paramagnetic resonance effect^19^. Disruption of the magnetic compass in presence of oscillating magnetic field has been suggested as a diagnostic tool for the radical pair reaction mechanism underlying the magnetic compass^15^. This effect was indeed experimentally observed in dozens of experiments performed by at least three groups independently in birds^20–24^ and in mammals^25^; this would seem sufficient to confirm RPM beyond reasonable doubt. It should however be noted that RPM fails to explain the obtained results *quantitatively*; the observed sensitivity thresholds of the magnetic compass to OMF in European robins and garden warblers are two orders of magnitude less than what the existing theory would give even with the most liberal choice of parameters^22,26,27^. In order to shed light on this controversial issue, which is however extremely important for understanding the magnetic orientation of birds, we conceived an experiment aimed at spatial localization of the compass receptor via its sensitivity to oscillating magnetic fields.

## Methods

**Figure 1:**
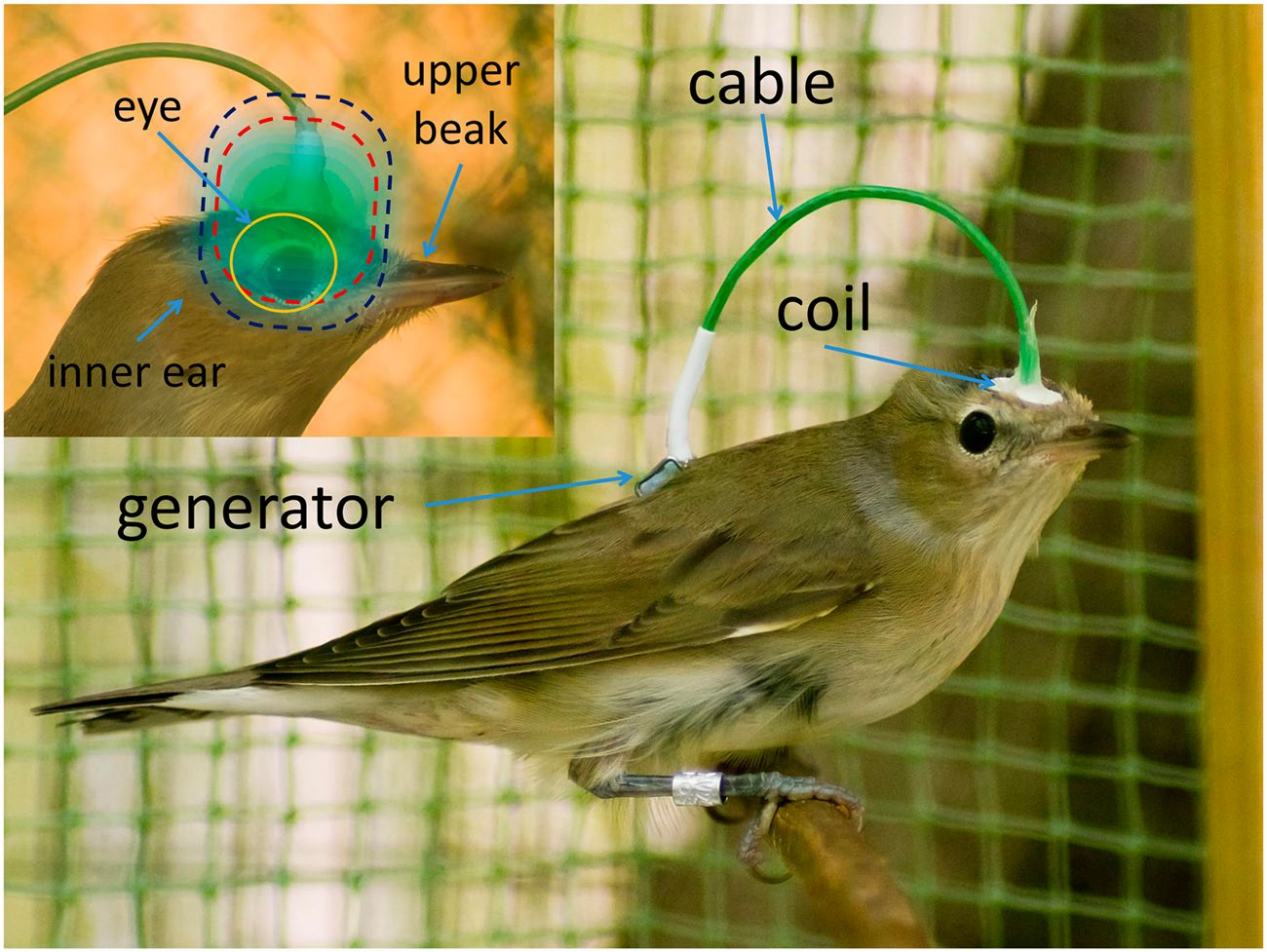
A garden warbler with attached portable device for local application of oscillating magnetic fields. *Inset*: The scheme of application of the oscillating magnetic field (OMF) to the head of a garden warbler. Blue and red dashed contours show, respectively, the boundaries of 2.5 nT and 5 nT OMF amplitude. Yellow circumference schematically shows the eyeball projection on the picture plane.

We developed miniaturized devices, each weighing just 0.95 g and comprising a magnetic coil and a high-frequency generator fed from watch batteries, which activated the coil at the frequency 1.403 MHz. Such a device (see SM for details) can be carried by a bird (garden warbler *Sylvia borin* in our experiments) with the coil attached to the bird’s head (Fig. 1). This way, we could create a volume of about 2 cm^3^ where the OMF amplitude exceeded 2.5 nT, i.e. it was larger than or approximately equal to the sensitivity threshold (2–3 nT) of the magnetic compass of garden warblers, determined in our earlier experiments^24^. This volume covered the retinae of both eyes (Fig. 1, inset). Notably, the OMF amplitude exceeded 5 nT in the central parts of the retina that form visual images. At the same time, the OMF in other parts of the birds head [upper beak and inner ear (lagena)], which might be involved in magnetoreception^28–32^, was considerably lower than the sensitivity threshold. In parallel, we performed standard experiments on disorientation of birds by 5 nT OMF created by large stationary coils and applied indiscriminately to the whole body of the bird.

We captured first-year garden warblers during autumn migration on the Courish Spit (Kaliningrad region, Russia; 55°09′N, 20°52′E). We kept the birds outdoors in individual wood and cloth-net cages (60 ×40 ×40 cm) for at least 5 days before the first test, to give them an opportunity to acclimate to cage conditions. The outdoor aviary was equipped with online video cameras, so that we could pick for experiments only the birds which showed nocturnal migratory restlessness. During captivity, the birds experienced natural photoperiod, local geomagnetic field conditions and had access to all astronomic orientation cues (sun, sunset polarization patterns and stars). The birds were tested in round arenas (Emlen funnels)^33^ placed inside a screened and grounded non-magnetic chamber in the laboratory house (see SM for details) with artificial nocturnal lighting by green LEDs. Under these conditions, with a frosted glass on the top of the Emlen funnels, the only orientation cue available to the experimental birds during the tests was the geomagnetic field. The birds were released back into the wild after all experiments had been completed.

Our experiments consisted of two phases. During the first phase, we worked with garden warblers without any devices on their bodies. Each bird was tested from one to four times until receiving the directional selectivity. During the second phase, birds passed three types of tests. They were tested with portable devices attached (Fig. 1), both in “on” and “off” state, which was achieved by equipping the mini-generators either by live watch batteries or by dummies of the same weight. The birds were also tested in a homogeneous 5 nT OMF (while not carrying the portable device, either before it was put on or after it had been removed) generated by stationary coils (see SM for details).

When birds with attached portable devices stayed in outdoors living cages, watch batteries were removed from the portable devices. Just before the test, watch batteries or dummies of the same weight were inserted into the sockets on mini-generators. We controlled the OMF generated by the coils just before and after tests, using a small ferrite antenna connected to a digital oscilloscope.

Each test lasted 50 min and usually started at the beginning of astronomical twilight. The directionality of the birds’ activity was recorded as scratches left by birds when they were hopping in the funnels on a print film covered with a dried mixture of whitewash and glue. Two researchers (AP and MErs or AP and MEro) independently determined each bird’s mean direction from the distribution of scratches. In most cases, we identified the mean direction using the simple visual estimation method^34^. If a pattern of scratches was not clear, scratches were independently counted in each of 36 ten-degree sectors and we used circular statistics to assess the directionality, based on the numbers of scratches^35^. The mean of the two observers’ determined directions was recorded as the orientation result. If observers considered the scratches to be randomly distributed or if the two mean directions deviated by more than 30°, the bird was considered to be disoriented in the given test. Inactive (fewer than 40 scratches) and disoriented birds were excluded from analysis. The group mean directions for each experimental condition were calculated based on individual mean directions. We included the results of all birds that were tested at least one time in experimental conditions, showed at least one sufficiently active result, and were significantly directed according to the Rayleigh test (at the 5% significance level^35^); (for details, see Table S1 of SM). A double-blind protocol was used in tests with portable devices: researchers who carried out behavioural experiments and quantified their results were not aware, whether the OMF mini-generator on a bird was turned on or turned off in each test, until the end of all experiments.

Out of 22 birds carrying the portable OMF device, 21 showed nocturnal migratory activity in the following nights. All tests were conducted during the first part of autumn migration of the species (August 23–September 16), to avoid the effect of the progress of season on orientation, found in garden warblers in our previous experiments^22,24^.

The differences in the mean orientation direction between birds in various experimental conditions were analysed using the nonparametric Mardia-Watson-Wheeler (MWW) test performed with ORIANA 4.02 (Kovach Computing Services, UK). We used the bootstrap technique^36^ to identify whether significantly oriented groups showed significantly more directed behaviour than non-statistically significantly oriented groups (see SM for details).

All animal procedures (in this case, capture of the birds and simple, non-invasive, behavioural experiments) were approved by the appropriate authorities: Permit 2017–24 by Kaliningrad Regional Agency for Protection, Reproduction and Use of Animal World and Forests; and Permit 2–2018 by the Bioethics Committee of Sechenov Institute of Evolutionary Physiology and Biochemistry RAS. All experiments were performed in accordance with relevant guidelines and regulations.

## Results

**Figure 2:**
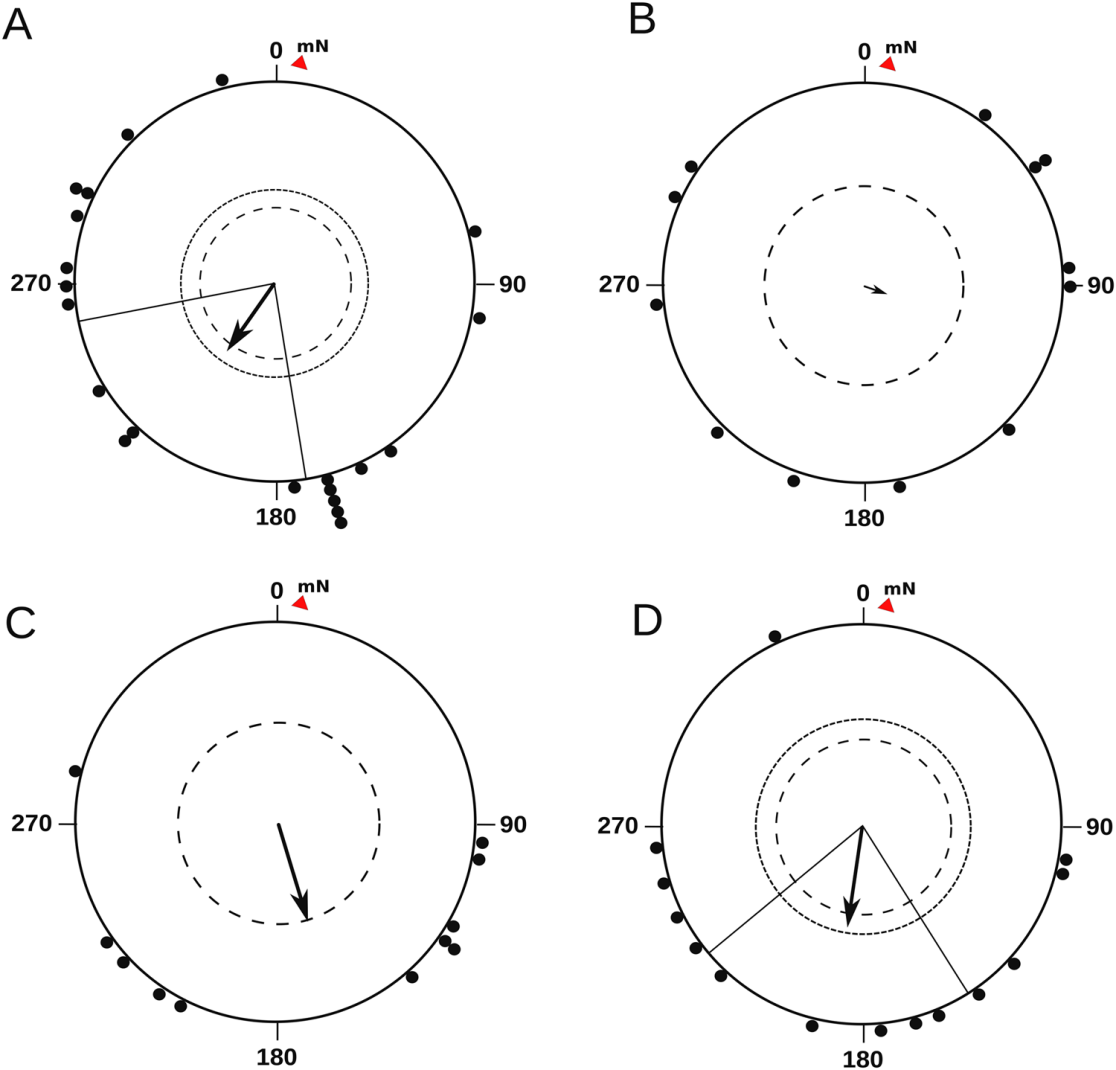
Orientation of garden warblers: (**A**) in the natural geomagnetic field (NMF); (**B**) in NMF plus homogenous 5 nT OMF generated by stationary coils at the frequency of 1.403 MHz; (**C**) in NMF with the portable device switched off; (**D**) in NMF with the portable OMF device switched on with local application of OMF to the bird’s eyes (as shown in the inset to Fig. 1). Dots show mean directions of individual birds in each experimental condition. Arrows show the second-order mean of the group of birds in each condition. The inner and outer dashed circles indicate 5 and 1% significance level of the Rayleigh test, respectively. Radial lines indicate 95% CI. The red triangle and letters mN are the position of the magnetic north.

Garden warblers, free of any devices on their bodies, showed appropriate migratory direction (Fig. 2A) in indoor conditions (α = 216°, n = 21, r = 0.38, P = 0.045). The mean direction of birds obtained indoors was similar to the mean autumn migratory direction of the same species, according to recoveries of birds ringed on the Courish Spit (α = 213°, n = 14, r = 0.96, p «0.001, 95% CI = 205°–222° ^37^; and unpublished data of the Biological Station Rybachy) and to the data obtained in previous experiments in Emlen funnels in garden warblers (α = 194°, n = 38, r = 0.41, p = 0.001, 95% CI = 169°–229° ^24^; these two distributions (in indoor and outdoor experiments) do not differ according to MWW test: W = 0.3, p = 0.86).

As we expected, the same birds, being subjected to homogeneous 5 nT OMF created by stationary coils, were disoriented (Fig. 2B; α = 106°, n = 12, r = 0.11, P = 0.86). The presence of portable devices on the body of a bird did not seem to affect its orientation behaviour. The mean orientation vector in birds carrying the switched off device was just below the significance level (α = 164°, n = 11, r = 0.51, P = 0.056; Fig. 2C). Extraordinarily, when the device was on, they were not disoriented either: the birds showed migratory orientation appropriate for autumn migratory season (α = 189°, n = 14, r = 0.49, P = 0.03; Fig. 2D).

The direction of activity shown by garden warblers with OMF devices switched on was statistically indistinguishable from the direction shown by the birds without devices (MWW test: W = 0.15, p = 0.93) and the birds with devices switched off (MWW test: W = 0.49, p = 0.78).

We additionally verified these results by using the bootstrap analysis, which confirmed that significantly oriented groups showed significantly more directed behaviour than non- significantly oriented groups.

## Discussion

Our experimental results have significant implications for the radical-pair-based compass sense, as well as for further search of the magnetic-compass receptor in birds. One can hypothesize three possible structures of the compass magnetoreception system that would not contradict to the results of our experiments:

1. *Compass sense is based on cryptochrome receptors in the retina, but its disruption by OMF is not a result of direct action of OMF on the compass receptor*. This hypothesis relieves the radical pair theory from the unresolved problem of quantitative explanation of the OMF effect. However, it raises new problems: another, so far unknown, receptor must exist, which is extremely sensitive to OMF, and the compass and OMF receptors must be neurophysiologically connected so that the OMF receptor could send a command which would block signals from the compass receptor.
2. *The compass receptor is not located in the eye*. Since radical pair reactions in cryptochrome require light for their initiation, this would most likely mean that this receptor works on some other physical principles. The most plausible alternative to RPM, suggested so far, is the magnetoreceptor based on nanoparticles of magnetite or some other magnetic material. According to available data, such a receptor might be situated either in the upper beak^28,38,39^, [but see^40^] or in the lagena^29–32^, [but see^41^], even though other locations cannot be definitely excluded. The main problem of this hypothesis is that no design of the magnetite-based receptor was put forward, which would account for extreme sensitivity of the bird’s compass to OMF.
3. *The cryptochrome-based compass magnetoreceptors are situated only in the ventral part of the retina*. Such receptors might be just marginally affected by OMF from our portable devices, since in the ventral part of the bird’s eye the OMF amplitude was close to the sensitivity threshold of 2–3 nT, determined earlier for the garden warbler. We consider this scenario unlikely. However, if this is true, it still means that the magnetic field direction is not perceived by the bird via patterns superimposed on visual images, since the central part of the retina was affected by OMF of 5 nT and stronger, which caused total disorientation of birds when applied to their whole bodies in our control experiments.

One may also suppose that the behaviour demonstrated by birds with switched on devices is not actually a true magnetic orientation. Several papers described directional orientation that did not originate in radical pair processes in the eyes and was not disrupted by RF-fields [e.g.^42–45^]. These so-called ‘fixed direction responses’ were reported when the radical pair mechanism was disrupted, and attributed to magnetite-based receptors in the beak. If this ‘fixed direction response’ existed in our case, and its direction by chance coincided with the S-SW migratory direction, it would result in the observed orientation pattern. However, following these logics, one would expect this ‘fixed-directional response’ to appear under global application of the OMF by large coils as well, which was not the case in our experiments. We can think of no reason why OMF generated by minicoils elicits fixed direction responses, whereas OMF generated by large coils does not. We therefore consider this explanation unlikely.

## Conclusions

Our experiments have demonstrated the insensitivity of the bird magnetic compass to oscillating magnetic fields applied locally to the eyes. This result does not necessarily deny the key role of radical pair reactions in magnetoreception. However, it definitely disproves the radical-pair model in its most ambitious form, which in particular suggests that: (i) magnetic sensitivity of photochemical reactions in cryptochrome molecules situated in the retina produces a visual image of the geomagnetic field, (ii) the compass is disrupted by OMF due to its effect on electron spins in radical pairs formed by the cryptochrome. Indeed, if it were so, the magnetic-field induced image would have been destroyed by the OMF that we applied to the central part of the retina. Our findings therefore point out to existence of other, so far unknown, components of the avian magnetoreception system.

## Supplementary information

Supplementary information.

## Data Availability

All data generated or analysed during this study are included in this published article (and its Supplementary Information files) or available from the corresponding author on reasonable request.
