# Supplementary material for: Magnetic compass of garden warblers is not affected by oscillating magnetic fields applied to their eyes
Source: Sci Rep. 2020 Feb 26;10:3473. doi: 10.1038/s41598-020-60383-x (PMC7044251; doi:10.1038/s41598-020-60383-x)
Supplement: Supplementary file 1 — Supplementary information. [file 41598_2020_60383_MOESM1_ESM.pdf]

Magnetic compass of garden warblers is not affected by oscillating magnetic fields applied to their eyes

Julia Bojarinova, Kirill Kavokin, Alexander Pakhomov, Roman Cherbunin, Anna Anashina, Maria Erokhina, Maria Ershova, Nikita Chernetsov

SUPPLEMENTARY MATERIALS

The device for local application of oscillating magnetic fields (OMF)

The portable device for local application of OMF comprised a miniaturized generator, an induction coil and a connecting cable (Fig. S1). We designed the radio-frequency generator as a feedback oscillator with an LC resonator tuned to 1.403 MHz. Miniaturized SMD generators were developed according to our circuit diagram and produced in a small series by Thirdpin (www.thirdpin.io/en/). The power supply was provided by two detachable watch batteries. The amplitude of the output current was set to 0.55 mA. The connection cable was an unshielded twisted pair of 0.3 mm copper wires in plastic insulation, 70 mm in length. Four loops of the same wire, 4 mm in diameter, formed the induction coil that had the figure 8 shape. The upper and lower parts of the figure 8 consisted of two round windings of the wire each. The current directions in the two parts of the figure 8 were opposite, so that it formed a magnetic quadrupole. This arrangement provided rapid diminishing of the OMF intensity with distance from the coil in the far-field zone. The coil was sandwiched between two pieces of thin paper, glued together, and fixed to the cable insulation with a drop of sealant. The total weight of the device with installed watch batteries was 0.95 g.

We fixed generator to the bird’s back by the method that is usually employed for fixing transmitters or loggers onto small birds, i.e. by using two leg-loops. The leg-loops were made of elastic sewing thread and adjusted to the size of the garden warbler. The coil assembly could then be glued to the paper patch previously fixed to the bird’s head by nontoxic eyelash glue (Professional makeup Duo eyelash adhesive). Before gluing the paper patch, the feathers on the bird’s heard were cut short with scissors. Birds could stay with attached devices for 5-7 days,

demonstrating normal behaviour and showing night migratory restlessness. After this period, the coil started to peel off the birds head, and we removed the device.

To calibrate the OMF created by this device, we measured the current in the coil and made sure that it was equal to the output current of the generator, i.e. there were no sizable current leakage in the coil assembly and in the twisted pair. The amplitudes of the OMF vector components as functions of coordinates were calculated from the coil current using the Bio-Savart law. The calculated spatial distribution of the OMF was verified by comparison with the measured electromotive force in a 2-mm probe coil connected through a broadband preamplifier to a lock-in amplifier (Standford Research SR844). The lock-in amplifier was set to the internal reference mode, which provided narrow-band detection at the fundamental frequency of the generator. The position of the probe coil was scanned using a 3D micrometric translation stage (Thorlabs). Figure S2 (A and B) shows measured profiles of two OMF components as functions of coordinates, in comparison with the calculated dependences. In Fig. S2 (C and D), calculated boundaries of the zones with the OMP intensity exceeding 2.5 nT (previously measured sensitivity threshold of the garden warbler magnetic compass) and 5 nT (the amplitude of homogeneous OMF in our experiments) are shown.

Figure S3 (A) shows the simulated distribution of the OMF intensity amplitude over the surface of the retina. The retina of a Garden warbler was approximated by a sphere 7mm in diameter, with the center situated 4.5 mm below the coil plane and 4 mm from the sagittal plane of the bird head. One can see that the OMF intensity amplitude exceeds the threshold value of 2.5 nT everywhere but the narrow strip in the ventral part of the retina. In the central and dorsal part of the retina it is be far stronger.

The same probe coil was used to measure the OMF frequency spectrum. To this end, the time-dependent signal from the preamplifier was sampled each 100 ns with a digital oscilloscope (Tektronix TPS 2012B), and the obtained time series (2500 readings each) were Fourier transformed and averaged over 30 repetitions. The resulted spectrum is shown in Figure S4.

The laboratory house

We have constructed a purpose-built wooden laboratory outside the main building of Biological Station Rybachy. It consists of two rooms: the biggest one (measurements 4 m x 4 m x 4 m) was used as a main experimental chamber, and its walls were plated with aluminium and grounded. During experiments, the conductive roller door of the chamber was lowered, closing the Faraday cage. All items of electric equipment (a power supply for green light inside the first chamber, a signal generator, etc.) used in our experiments were placed in the second small chamber and also grounded. The experimental chamber was illuminated with dim green light (with a peak wavelength of 527 nm) produced by light-emitting diodes (LEDs). The intensity of the light within the funnel cages under with the milky-glass was 0.25 ± 0.05 mW/m^2. LED tapes were mounted on an aluminium frame (under the chamber ceiling) and fed by a programmable DC power supply Rigol DP711 (Rigol Technologies, Inc., Beaverton, USA). We used an optometer P-9710 (Gigahertz-Optik GmbH, Germany) to check the light condition before or after tests. The spectrum of illumination was measured by a handheld spectrometer UPRtek MK350N (UPRTEk Corp., Taiwan).

Stationary coils and Emlen funnels

The stationary coils were the same ones that were described in our previous study [22]. A signal generator Rigol DG4162 (Rigol Technologies, Inc., Beaverton, USA) produced oscillating current to feed coils. We used narrow-band RF fields with a frequency of 1.403 MHz, matching the Larmor frequency of a free-standing electron spin in the local geomagnetic field of 50 100 nT, and with an amplitude of 5 nT. The parameters of RF field produced by stationary coils were measured and controlled by a 25 cm loop antenna, connected to a digital storage oscilloscope Tektronix TPS 2012B (Tektronix, Inc., USA), before and after each test.

We carried out all the orientation tests, except those with OMF applied using stationary coils, in modified Emlen funnels [33] made of aluminium (top diameter 300 mm, bottom diameter 100 mm, slope 45°). Additionally, each funnel was placed inside a high plastic cylinder with the top covered by netting to prevent contacts between the mini-generators and netting, that could cause disturbance of the bird and/or generator damage. All tests in stationary OMF coils were performed only in funnels made of a dielectric material (plastic), to avoid the screening effect of aluminium Emlen funnels on the radio-frequency field [24]. On tops of the plastic funnels and cylinders, we put lids made of frosted glass which completely obscured all environmental cues in the wooden house but let enough light in.

Bootstrap technique

We used the bootstrap technique [36] to identify whether significantly oriented groups showed significantly more directed behaviour than non-statistically significantly oriented groups. According to the method that was described in previous studies [S1,S2], a random sample of orientation directions (for example, $n = 12$, it was equal to a number of birds in OMF stationary coils) was drawn with replacement from the sample of orientation directions present in the significantly oriented group (birds with switched on devices, $n = 14$). Based on these 12 orientation angles, the corresponding r-value was calculated, and this procedure was repeated 100 000 times. After that, the resulting 100,000 r-values are ranked in ascending order. The r- values at rank 2500 and 97500, at rank 500 and 99500 define the 95% and 99% limits for the actually observed r-value of the significantly oriented group, respectively. If the actually observed r-value of the disoriented group (for example, birds in OMF stationary coils) lies outside these confidence intervals, the significantly oriented group (for example, birds with switched on devices) is significantly more directed than the non-significantly oriented group with a significance of $p < 0.05$ and $p < 0.01$, respectively.

Comparison of OMF created by portable and stationary coils

1) *Frequency spectrum.*

As expected, spectral characteristics of our miniaturised generators are inferior to

those of laboratory generators. As seen from Fig.S3, the amplitude of the 3rd harmonic is about 0.15, and the amplitude of the background noise is about 0.01 of the 1st harmonic amplitude. To compare, the same characteristics of the generator (Rigol DG4162) that we used to feed stationary OMF coils are approximately 0.003 and 0.0001, respectively [24]. Since higher sensitivity to broadband magnetic noise than to monochromatic OMF was reported earlier for European robins [23, S3], one could expect the noisy OMF produced by mini-generators to exert even stronger effect on birds' magnetic compass than highly monochromatic fields of stationary coils with the same amplitude and frequency of the main harmonic. Therefore, spectral characteristics of our portable OMF devices cannot account for the absence of disorientation effect.

2) *OMF alignment with respect to the geomagnetic field.*

One can argue that the OMF applied with mini-coils, as distinct from one applied with stationary coils, has fixed direction with respect to the bird head. Therefore, the bird can turn its head in such a way as to make the OMF parallel to the static geomagnetic field; in this arrangement of fields, the absence of the OMF effect in European robins was reported [20]. However, as seen from Fig. S2 (A and B), the vector components of OMF created by mini-coils strongly depend on coordinates, so that it is directed differently at different points of the retina. Therefore, the parallel-fields arrangement cannot be realized for the retina as a whole; it is possible only for certain points, positions of which on the retina would depend on the angle of the bird head. This is illustrated by Fig. S3 (B), showing the OMF component perpendicular to the geomagnetic field (NMF) as a function of the position on the retina. In our previous experiments, Garden warblers were disoriented when OMF amplitude was higher than 2.5 nT; in those experiments, the OMF comprised the angle of 30 degrees with the NMF, so that the minimal perpendicular component of OMF was 1.25 nT. As seen from Fig. S3 (B), such low values of the perpendicular OMF are realized only within very small areas on the retina; this is due to the fact that the NMF is nearly vertical, while our mini-coil system produces OMF with a

strong horizontal component. Even if some receptors continued to work under such conditions, the information output from the magnetic compass would be entirely different from that in the absence of OMF. As orientation of garden warblers in our experiment demonstrated no statistical difference in presence of OMF created by portable devices and in its absence, we consider this explanation very unlikely.

Supplemental References

S1. Chernetsov, N. *et al*. Migratory Eurasian reed warblers can use magnetic declination to solve the longitude problem. *Curr. Biol*. 27, 2647–2651 (2017). doi:10.1016/j.cub.2017.07.024

S2. Alert, B., Michalik, A., Thiele, N., Bottesch, M. & Mouritsen, H. Re-calibration of the magnetic compass in hand-raised European robins (*Erithacus rubecula*). *Sci. Rep*. **5**, 62 (2015). doi:10.1038/srep14323

S3. Schwarze, S. *et al.* Weak broadband electromagnetic fields are more disruptive to magnetic compass orientation in a night-migratory songbird (*Erithacus rubecula*) than strong narrow-band fields. *Front. Behav. Neurosci*. **10**, 55 (2016). doi:10.3389/fnbeh.2016.00055

Supplemental Figure Legends

Figure S1. The portable device for local application of OMF. 1– generator; 2 – batteries; 3 – connecting cable; 4 – coil; 5 – leg-loops for fixation on the bird's back

Figure S2. (A) Lateral profile of the X-component of the OMF at different distances from the coil plane; (B) Lateral profile of the Y-component of the OMF at different distances from the coil plane; (C) Boundaries of 2.5 nT amplitude of OMF intensity in YZ planes at different X; (D) Boundaries of 5 nT amplitude of OMF intensity in YZ planes at different X. The inset shows the coordinates axes positioning with respect to the coil.

Figure S3. (A) Simulated amplitude of OMF intensity on the surface of the retina. θ is the angle to the eyecup axis, perpendicular to the sagittal plane. ϕ is the azimuthal angle in the sagittal plane. (B) Amplitude of the OMF component, perpendicular to the geomagnetic field (NMF) at our experimental site (inclination 70.1^{o}), on the retina surface for the bird heading to magnetic south, south-west and west.

Figure S4. The frequency spectrum of the OMF created by the portable device, normalized to the 1st harmonic amplitude.

Table S1. Orientation of each garden warblers in different experimental conditions: **NMF** – natural geomagnetic field only; **Gen on** – natural geomagnetic field + portable device switched on; **Gen off -** – natural geomagnetic field + portable device switched off; **OMF** – natural magnetic field + 5 nT oscillating magnetic field, without any devices. NA – not active, NS – not significant orientation behaviour.

**№**	**№ test** / **№ ring**	**NMF**					**Gen on**			**Gen off**				**OMF**		
		**1**	**2**	**3**	**4**	**Mean**	**1**	**2**	**Mean**	**1**	**2**	**3**	**Mean**	**1**	**2**	**Mean**
**1**	**20477**	240°				**240°**	255°		**255°**	NA	95°		**95°**	NA	NS	
**2**	**20500**	289°				**289°**	195°	NA	**195°**	285°			**285°**	NS	NS	
**3**	**20423**	165°				**165°**	131°		**131°**	228°			**228°**	225°	NS	**225°**
**4**	**20523**	315°				**315°**	335°		**335°**	NA	NA			NS	NS	
**5**	**20480**	NA	NA	175°	180°	**178°**	245°		**245°**	NS				NS	90°	**90°**
**6**	**20469**	NS	145°			**145°**	NA	101°	**101°**	NS	215°		**215°**	NS	NA	
**7**	**20459**	275°				**275°**	NA	165°	**165°**	NA				NA	NS	
**8**	**20470**	NA	NA	225°		**225°**	NS	NS		NA	139°		**139°**	55°	NS	**55°**
**9**	**20741**	NS	165°			**165°**	NA	NA		125°			**125°**	265°	NS	**265°**
**10**	**20623**	NS	165°			**165°**	235°		**235°**	NA	208°		**208°**	35°	75°	**55°**
**11**	**20599**	NS	165°			**165°**	225°		**225°**	120°			**120°**	NS	NA	
**12**	**20742**	265°				**265°**	NA	NA		NA	100°		**100°**	NA	305°	**305°**
**13**	**20689**	155°				**155°**	NA	NS		NA	235°		**235°**	NA		
**14**	**20744**	NS	165°			**165°**	175°		**175°**	NA	NA	NA		230°	170°	**200°**
**15**	**20638**	NS	NS	295°		**295°**	145°		**145°**	NS	NS	NS		170°	NS	**170°**
**16**	**20776**	NS	295°			**295°**	158°		**158°**	NS				35°	NS	**35°**
**17**	**20588**	NA	NA	NS	270°	**270°**	265°		**265°**	125°			**125°**	NA		
**18**	**20798**	NA	345°			**345°**	100°		**100°**	NS						
**19**	**20779**	NS	75°			**75°**	NA							295°	NS	**295°**
**20**	**20821**	NS	225°			**225°**								85°	NS	**85°**
**21**	**22242**	99°				**99°**								130°	NS	**135°**

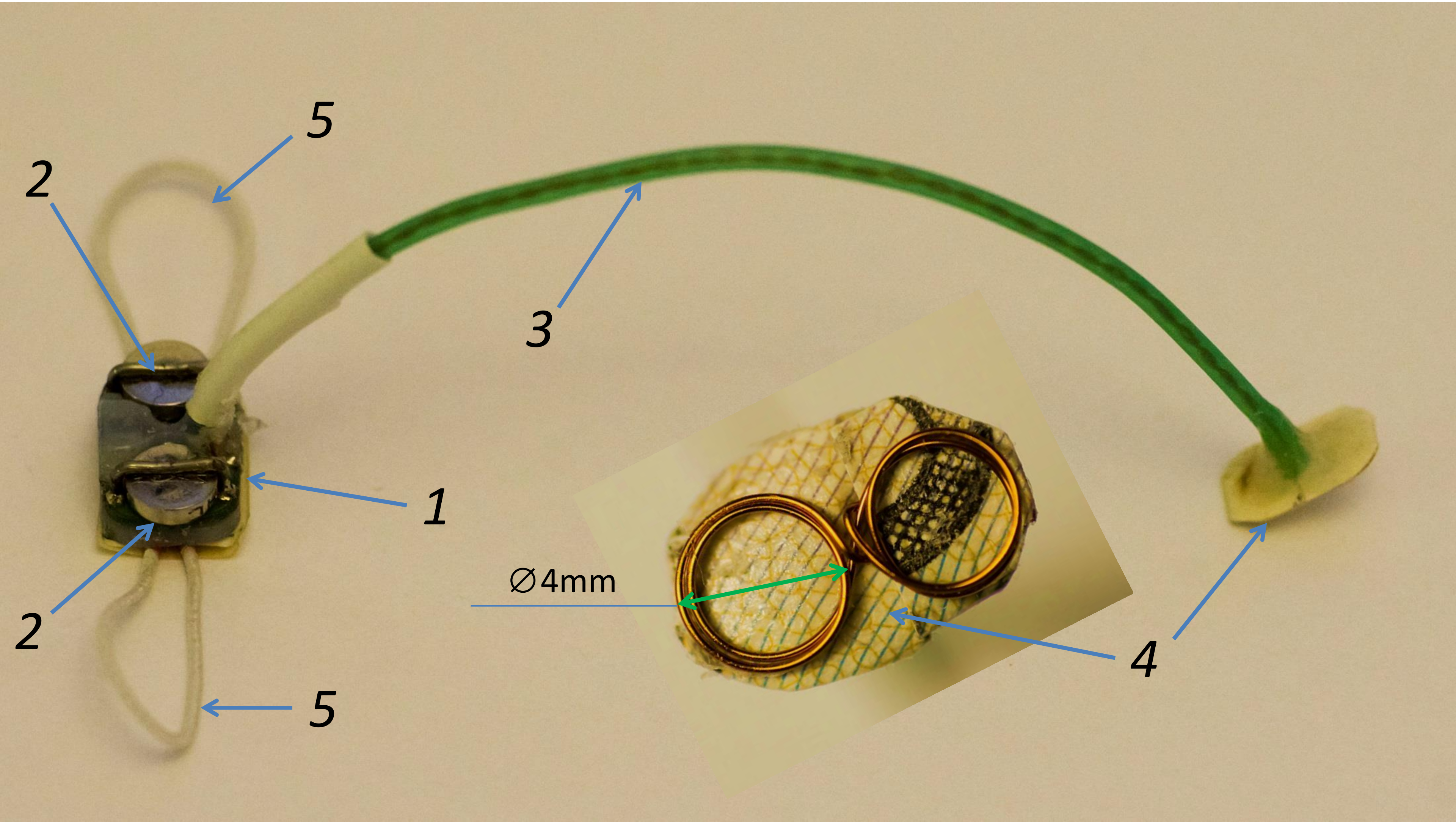

Figure S1

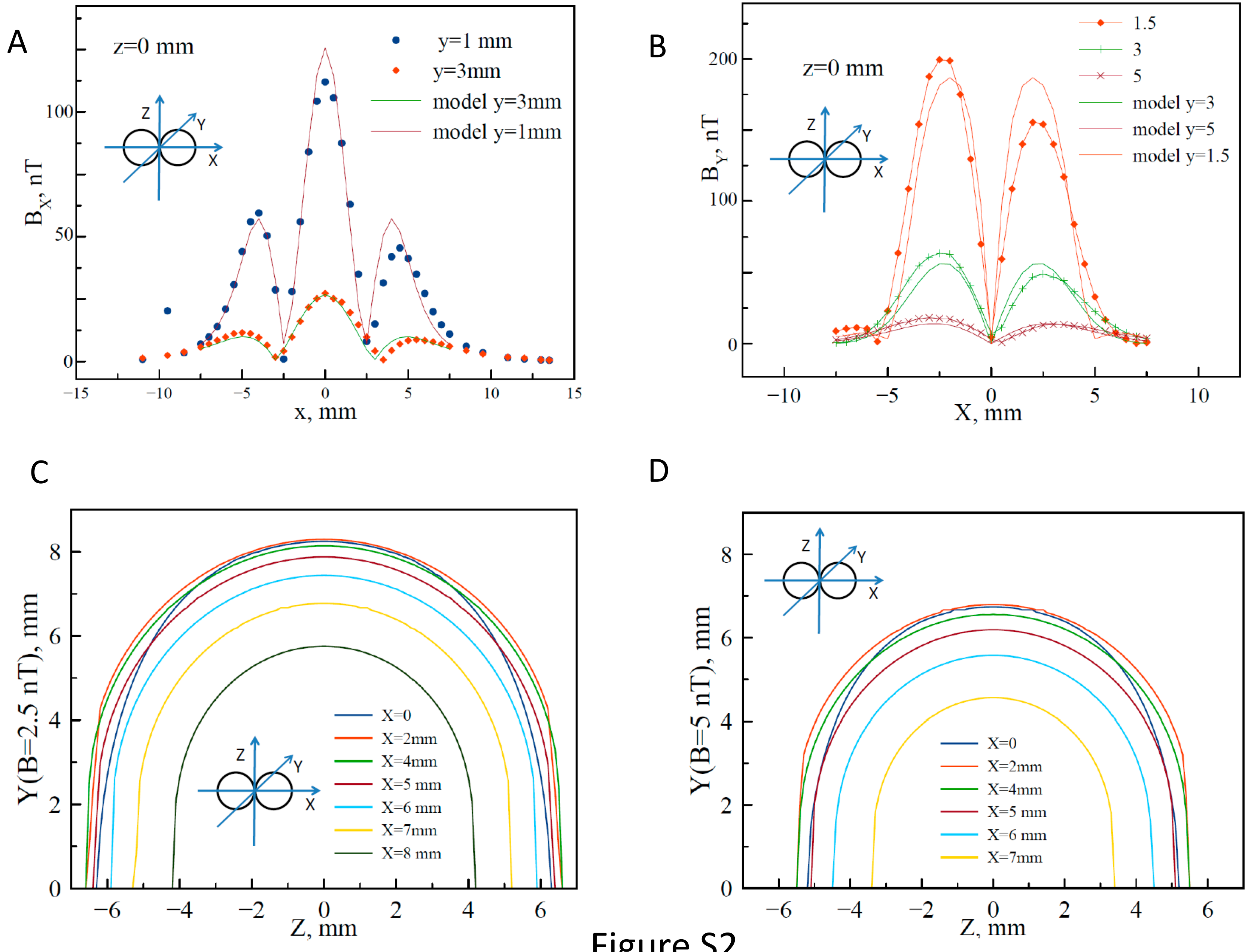
A
z=0 mm
y=1 mm
y=3mm
model y=3mm
model y=1mm
Z
Y
X
B_X, nT
x, mm
B
z=0 mm
1.5
3
5
model y=3
model y=5
model y=1.5
B_Y, nT
X, mm
C
Y(B=2.5 nT), mm
Z, mm
X=0
X=2mm
X=4mm
X=5 mm
X=6 mm
X=7mm
X=8 mm
D
Y(B=5 nT), mm
Z, mm
X=0
X=2mm
X=4mm
X=5 mm
X=6 mm
X=7mm

Figure S2

Figure S3

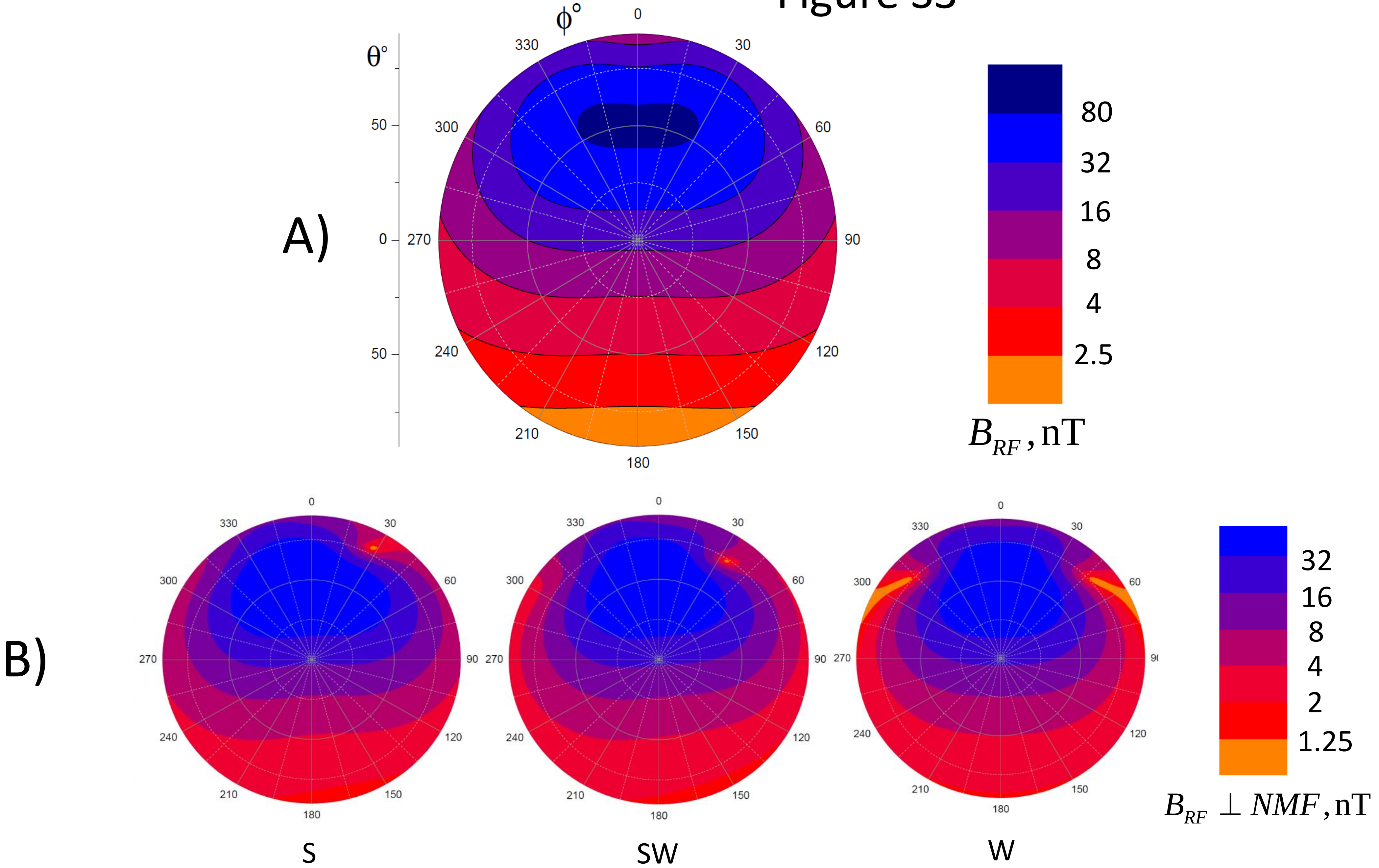

A)
φ°
θ°
0
30
60
90
120
150
180
210
240
270
300
330
50
0
50
80
32
16
8
4
2.5
B_{RF}, nT
B)
S
SW
W
32
16
8
4
2
1.25
$B_{RF} \perp NMF$, nT

Figure S4

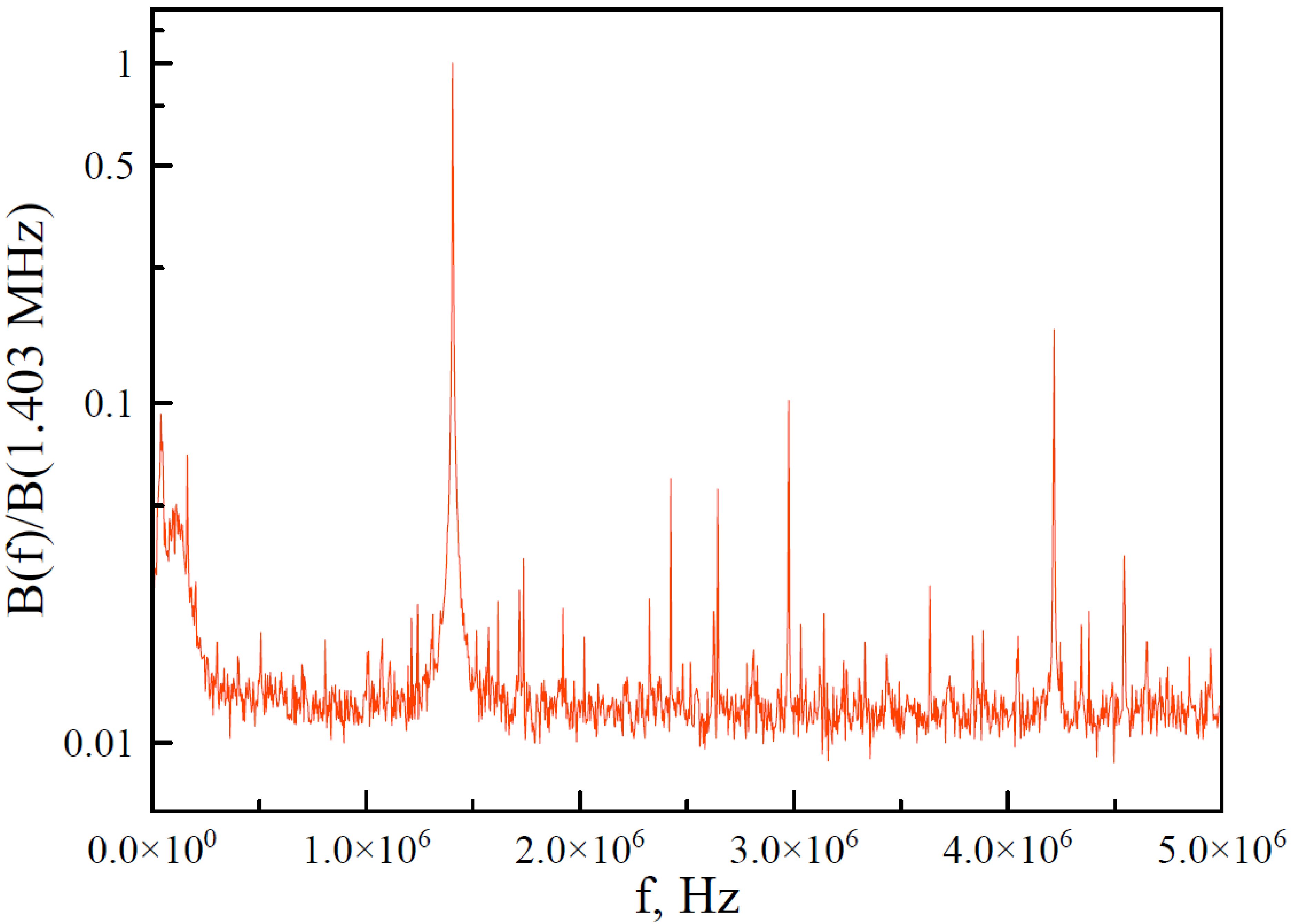
1
0.5
0.1
0.01
B(f)/B(1.403 MHz)
0.0×10^0
1.0×10^6
2.0×10^6
3.0×10^6
4.0×10^6
5.0×10^6
f, Hz
